# The Memories of NK Cells: Innate-Adaptive Immune Intrinsic Crosstalk

**DOI:** 10.1155/2016/1376595

**Published:** 2016-12-19

**Authors:** Sara Gabrielli, Claudio Ortolani, Genny del Zotto, Francesca Luchetti, Barbara Canonico, Flavia Buccella, Marco Artico, Stefano Papa, Loris Zamai

**Affiliations:** ^1^Department of Biomolecular Sciences, University of Urbino “Carlo Bo”, Urbino, Italy; ^2^IRCCS Istituto Giannina Gaslini, Genova, Italy; ^3^Department of Sensory Organs, University of Rome La Sapienza, Italy; ^4^Gran Sasso National Laboratory, INFN, Assergi, L'Aquila, Italy

## Abstract

Although NK cells are considered part of the innate immune system, a series of evidences has demonstrated that they possess characteristics typical of the adaptive immune system. These NK adaptive features, in particular their memory-like functions, are discussed from an ontogenetic and evolutionary point of view.

## 1. Introduction

Innate immunity and adaptive immunity are two complementary aspects of the immune response, which consists of a complex system of interactions aimed either at the clearance of pathogens or at the elimination of transformed cells. Although the two terms, inherited from the biology of the last century, present themselves as antithetical, the high degree of cooperation and interdependence between immunological mechanisms makes a clear-cut distinction between the two concepts increasingly difficult. This difficulty is also caused by the fact that, from a phylogenetic point of view, the adaptive immunity can be considered an evolution and an improvement of the innate immunity, and the coexistence of partially similar and mutually intertwined mechanisms is therefore possible [1].

The lymphoid cells which are traditionally considered as the effectors of innate immunity are NKT cells and the so-called ILC (innate lymphoid cells), among which NK cells are one of the most important populations [2]. They recognize their ligands in a non-MHC restricted way and, as part of the innate immunity, are generally considered unable to remember antigens and to increase the magnitude of their response over time [3]. The lymphoid cells which are traditionally considered as the effectors of adaptive immunity are T and B lymphocytes. They feature both the need to recognize the antigen and the ability to arouse a faster and stronger response when they encounter their cognate antigen for a second time. The two mechanisms underlying this last function, which is called immunologic memory, are basically the same in both populations. The first consists of an antigen-dependent clonal proliferation, and the second of a capability to maintain for a very long time (sometimes throughout life) a population of derived cells able to proliferate again on the occasion of a further encounter with the same antigen.

In T cells the process of memory formation can be divided into 3 distinct phases [4]. Initially there is a phase of “expansion” during which clones of naive T cells expand and differentiate into effector T cells following exposure to foreign antigens in the framework of the major histocompatibility system (MHC); this is followed by a phase called “contraction,” during which most effector T cells undergo apoptosis. Only a few cells survive and enter the third stage, called “memory,” during which they tend to persist and self-renew, ready to encounter the same antigen to which they had been previously exposed [5]. The receptor that plays a key role in the activation of the transcriptional program of naive T cells towards the formation of memory T cells is the T cell receptor or TCR [6], whose rearrangement during the maturation in the thymus allows recognition of any virtually possible antigenic determinant.

Gene rearrangement and the establishment of a memory cell population are features which are shared by B lymphocytes, whose antigen receptor (BCR) consists in membrane-bound immunoglobulins. After the first encounter with the antigen, B lymphocytes proliferate as well, giving rise to expanded populations of cells sharing the same antigen receptor. Compared to T lymphocytes, B lymphocytes are characterized by the presence of further mechanisms aimed at improving the efficacy of the response. The first mechanism is called somatic hyper mutation process (SHM) and consists in a modulation of the antigen receptor affinity by inserting random point mutations in the sequences coding for the variable regions of the receptorial immunoglobulin. Such editing, which takes place in the germinal center, is followed by the positive selection of the cells which display an improved antigen recognition [7]. The second mechanism, also carried out in the germinal center, is called isotype-switching or class-switch recombination (CSR). According to CSR, postgerminative B cells and related plasma cells mount and produce immunoglobulins characterized by a new constant region. When secreted, these isotype-switched immunoglobulins are able to perform different biological activities, aimed at a more rapid clearance of pathogens in the different contexts in which they are released [7]. Irrespective of the different strategies put in place to ensure the presence of long-lived populations, or to improve their response, there is no doubt that the effectiveness of the immunologic answer relies on the gene rearrangement able to amplify the repertoire of receptors.

It is interesting to note that the amplitude of the rearrangement depending repertoire is not the same in every cell population able to rearrange. It is similar in B and alpha/beta T lymphocytes, but it appears significantly reduced in gamma/delta T lymphocytes, which are considered as a phylogenetically older population [1], specialized in the immediate recognition of a reduced range of pathogens [8]. From this point of view, gamma/delta T lymphocytes, as well as NKT cells, which are traditionally included in the innate immunity mainly because their effector activity is independent of MHC, are actually located in an overlapped gray area between innate and adaptive immunity [9]. They further differ from other effectors characterized by the expression of receptors not subjected to rearrangement processes. NK cells belong to this latter group.

## 2. Natural Killer Cells: Part of Innate Immunity

Natural Killer (NK) cells were initially described for their functional ability to kill cancer cells without prior sensitization. For this feature, they have been always counted within the innate immunity. They represent 10–15% of circulating lymphocytes which are able not only to lyse tumor and pathogen-infected cells, but also to secrete cytokines. NK cells respond to both cytokine stimulation and cell to cell contacts with fast kinetics, resulting in strong effectors when B and T cells, which recognize specifically foreign antigens, are still in limited number or absent.

**Figure 1:**
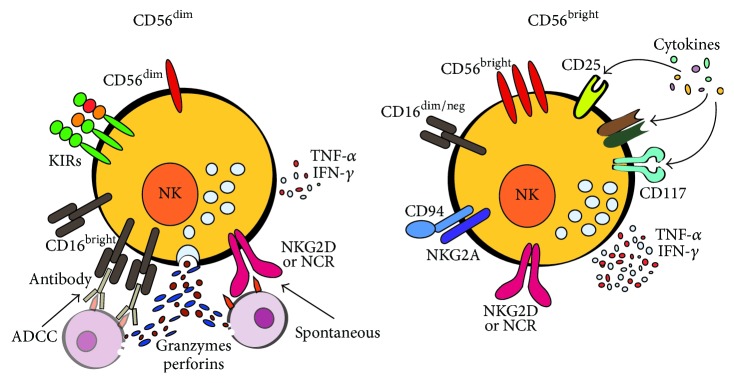
NK cell subsets. The majority of peripheral blood human NK cells belongs to the highly cytotoxic CD56^dim^/CD16^bright^ NK subset, while the “cytokine producers” CD56^bright^ NK cells are more abundant in secondary lymphoid tissues. Some differences in the receptor expression and response to stimuli are indicated; remarkably, the level of CD16 expression has functional consequences for the antibody-dependent cell cytotoxic (ADCC) mechanism.

Human NK cells are a heterogeneous population of cytotoxic cells that can be divided into two main subsets, based on the different density of expression of CD16 and CD56: CD56^dim^/CD16^bright^ and CD56^bright^/CD16^dim/neg^ [10]. Both NK populations can mediate cytotoxicity and secrete cytokines, but, similar to CD8 and CD4 T cells, they have significant phenotypic and functional differences (Figure 1). CD56^dim^/CD16^bright^ NK cells represent about 90% of NK cells in peripheral blood, and they show a low expression of CD56 and a high expression of CD16 and the human killer cell immunoglobulin- (Ig-) like receptors (KIRs); functionally they perform strong antibody-dependent cell cytotoxicity (ADCC) and spontaneous cytotoxic activity, preferentially against cells of hematopoietic origin. CD56^bright^ NK cells show a high expression of CD56 and CD94/NKG2A antigens; functionally they have a high ability to produce immunoregulatory cytokines, in particular interferon- (IFN-) *γ* and tumor necrosis factor- (TNF-) *α*. CD56^bright^ and CD56^dim^ NK cells have been proposed to represent either different NK cell stages or distinct NK subpopulations [10–12]. Consistent with this latter hypothesis are the evidences of their different pathways of in vitro differentiation [12–14] and responses to stimuli. In fact, CD56^bright^ and CD56^dim^ NK cells are preferentially activated by NK activating cytokines or by cell targets, respectively [15, 16].

It is known that NK cells develop, in both mice and humans, in the bone marrow. Nevertheless, NK cell progenitors can be also found in secondary lymphoid and peripheral organs, suggesting that they can leave bone marrow and differentiate in other locations [17–19]. NK cell maturation is accompanied by the expression of a number of activating and inhibitory receptors in a fashion that prevents NK-mediated autoaggression against normal cells [20]. Indeed, inhibitory and activating receptors finely tune NK cell functional activities. Among several inhibitory receptors expressed by NK cells, those sensitive to MHC class I (MHC-I) molecules expressed on target cells are of particular interest. From an evolutionary point of view, the lectin-like CD94/NKG2(A,B) heterodimeric receptors are the oldest; in fact they are expressed on both human and mouse NK cells. These receptors bind to human HLA-E or mouse Qa-1, whose expression on the cell surface depends on loaded peptides deriving from MHC-I molecules [21]. By a phylogenetic point of view, the lectin-like Ly49 receptor family in mice and the human killer cell immunoglobulin- (Ig-) like receptor (KIR) family are more recent. In fact, despite their similar function of recognizing MHC-I molecules, they have different structures, suggesting that the genes evolved after the divergence of the two species. These receptors sense overall MHC-I expression on target cells and eventually block activating signals; intriguingly, activating forms of KIRs, Ly49, and lectin-like CD94/NKG2(C,E,H) have been also described [20, 21]. The MHC-I sensing receptors transmit inhibitory signals which spare normal self-cells, but on the other hand they allow increasing the cytotoxic potential of NK cells that express them, forming the molecular basis of the “missing self” recognition [22]. As a matter of fact, the MHC-I-specific inhibitory receptors regulate not only NK cell reactivity against targets, but also their overall “licence” to kill target cells in a missing self-MHC-I fashion [23, 24]. Of note, some allele of KIR-HLA-I pairs seems to protect from HIV [25, 26], suggesting that KIRs, differently from CD94/NKG2(A,B) receptors, could represent a new immune armament against recently evolved diseases. Several receptors have been described to participate in the spontaneous NK cell activation, among which are NKp46, NKp30, and NKp44 (collectively termed Natural Cytotoxicity Receptors, NCRs), NKG2D, DNAM-1 (CD226), 2B4 (CD244), LFA-1 (CD11a-CD18), and CD2 [27]. Some of these act as costimulatory receptors that synergize with the real triggering receptors. The most important activating receptors responsible for NK spontaneous cytotoxicity are NCRs and NKG2D, which, by engaging their ligands expressed on the surface of “stressed” cells in the absence of inhibitory signals, lead to NK cell activation and target cell death [20, 28]. Another important activating receptor is represented by CD16 (or Fc*γ*RIIIA), the low affinity receptor for immunoglobulin G (IgG), able to bind immune complexes and IgG-coated, opsonized cells. Binding of this receptor with the crystallisable fragment of IgG on the opsonized target cells activates CD56^dim^/CD16^bright^ NK cells to kill through ADCC mechanism and to produce cytokines (see Figure 1).

**Figure 2:**
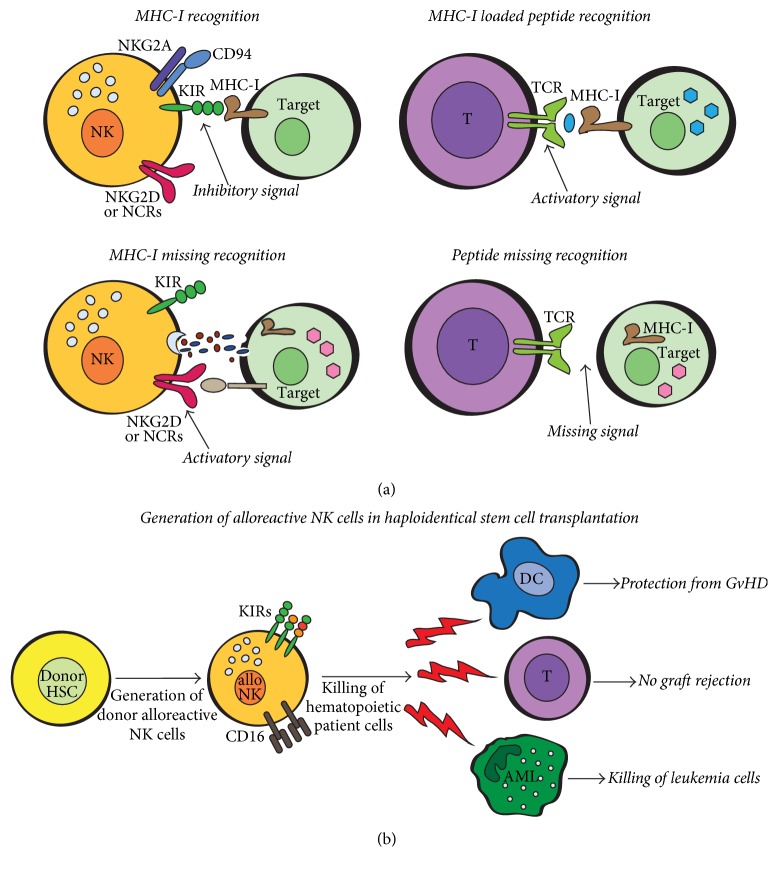
(a) MHC class I-driven mechanisms of recognition by NK and T cells. T lymphocytes and NK cells display complementary reactions in response to MHC-I surface expression on target cells. MHC-I loaded with (foreign) peptides are necessary to bind to T cell receptor (TCR) and to activate T cells. On the contrary, the engagement of HLA-I molecules with NK MHC-I sensing inhibitory receptors (KIR and/or CD94/NKG2A) blocks NK cell responses. When NK activating receptors (NKG2D and/or NCRs) recognize their ligands on surface of target cells that lack MHC-I molecules (self-MHC-I missing recognition), NK cells kill, releasing granzymes and perforins. (b) Exploitation of NK cell missing self-recognition in the improvement of haploidentical hematopoietic stem cell transplantation. Hematopoietic stem cells (HSC) from a selected donor can generate alloreactive CD56^dim^ NK cells which are able to kill not only the patient's neoplastic cells, but also patient dendritic cells (DCs) and T lymphocytes, reducing leukemic relapse, graft versus host disease (GvHD), and graft rejection.

Interestingly, NK cells play a complementary role to CTLs in MHC-I-driven immune responses: MHC-I molecules loaded with peptides are necessary to activate T cells, while they inhibit NK cells (Figure 2(a)). Of note, missing self-recognition of NK cells can be exploited in the improvement of haploidentical hematopoietic stem cell transplantation [29–32]. In fact, a haploidentical donor (usually a relative), who expresses the class I groups which are missing in the recipient, mediates NK cell killing of recipient leukemic, dendritic, and T cells (alloreaction) which reduces leukemic relapse, graft versus host disease (GvHD), and graft rejection (Figure 2(b)).

## 3. First Evidences of NK Adaptive Features

Although NK cells are considered innate immune cells, the fact that NK cells possess a number of characteristics in common with the cells of the adaptive immune system is emerging. NK cells share, in particular with T cells, not only the common lymphoid and the bipotent T/NK progenitors [33, 34], but also a series of functional features. In fact, NK cells have been described to be activated by dendritic cells [35], involved in autoimmune response [36], and able to recognize and respond to viral peptides [37, 38]. In particular, it has been shown that some MHC-I loaded viral peptides can antagonize inhibitory signals from KIRs, possibly masking KIR-mediated self-recognition. This last observation suggests that, in analogy to the T cell thymic maturation, a selection of KIR-HLA-I pairs able to “recognize” self-antigens should sometime occur during ontogenesis or phylogenesis. Evidences that some combinations of maternal KIR and fetal HLA-I molecules influence the reproductive success [39] suggest the possibility that, along the evolution of the human species, maternal uterine KIR^+^ NK cells could have biased the HLA-I (loaded with fetal peptides) of newborn. Of note, the too low- or too high-affinity binding of maternal KIRs to fetal MHC class I molecules is related to the birth of babies too small or too large, who are less likely to survive, a process resembling the outcome of thymic selection dependent on binding affinity of TCR for self-peptide-MHC complexes [40, 41].

**Figure 3:**
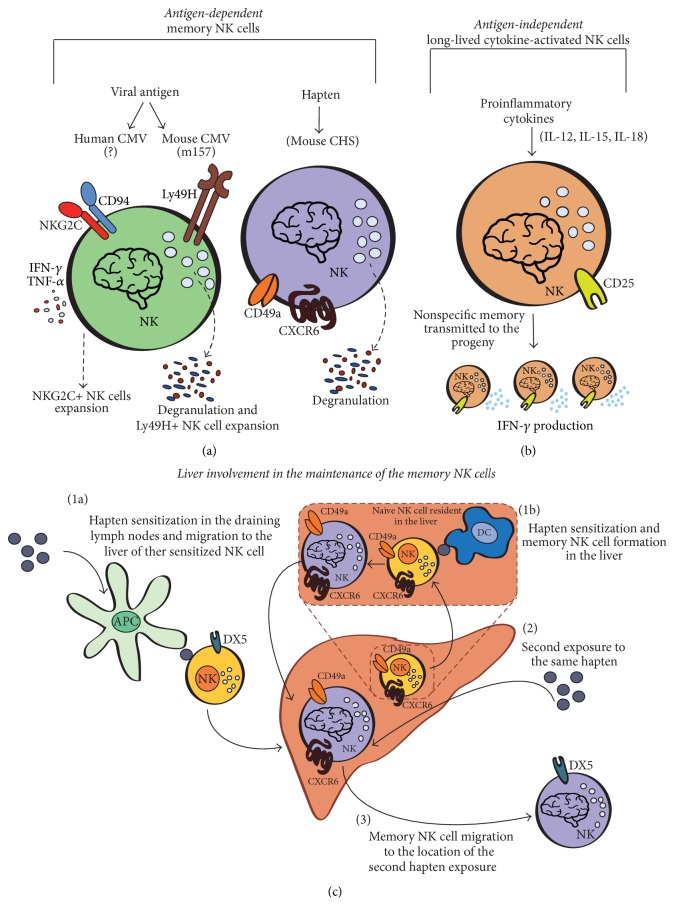
Memory-like NK cell formation. (a) Antigen-dependent memory-like NK cells. During CMV infection, NK cell memory is generated. In mouse it occurs through the recognition of MCMV m157 protein by the activating Ly49H receptor, while in human the ligand involved has not been identified yet; nevertheless, HCMV infection is associated with the expansion of CD94/NKG2C^+^ NK cell population. Hapten-specific memory-like NK cells are generated in the liver of a mouse model of hapten-induced contact hypersensitivity (CHS). Among the hepatic NK cell populations, CD49a^+^/DX5^−^ NK cells confer hapten-specific CHS memory-like responses, while the expression of CXCR6 is believed to have a role in their liver homing. (b) Antigen-independent long-lived cytokine-activated NK cells. They are generated after exposure to cytokines (IL-12, IL-15, and IL-18) and are characterized by prolonged longevity, CD25 expression, and intense IFN-*γ* production upon restimulation. (c) Hepatic localization of memory-like NK cells in CHS model. (1a) NK cells are activated in draining lymph nodes by antigen presenting cells, and newly generated memory-like NK cells move into the liver. Alternatively, (1b) antigenic recognition by NK cells directly occurs in the liver. (2) Second exposure to the hapten activates CD49a^+^/DX5^−^ memory-like NK cells and (3) migration of CD49a^−^/DX5^+^ NK cells into organs where the hapten is present.

Finally, NK subpopulations are stably expanded in response to some viral infections or cancers and seem to be involved in the long lasting control of these diseases [25, 26, 42–44]. Altogether, these findings have highlighted that NK cells possess adaptive features and have suggested their memory-like activity. This last characteristic has been observed for the first time in a mouse model of hapten-induced contact hypersensitivity (CHS) [45]. In Rag2-deficient mice, lacking both B and T cells, NK cells are necessary and sufficient to mediate contact hypersensitivity and hapten-induced memory [46] (Figure 3(a)). Although the molecular basis for the specificity remains unclear, NK cell-mediated CHS is strictly antigen-specific, since different haptens used for restimulation did not produce a memory-like response [47]. Adoptive NK cell transfer from donor liver, but not from other donor's districts, is itself capable of transferring memory to a naive recipient, suggesting that memory-like NK cells are localized in the liver [46, 47]. These memory-like NK cells are characterized by expression of CXCR6 chemokine receptor, and the need of its presence on NK cell surface for mediating CHS has suggested a role in their liver homing and the importance of their liver localization [47]. Indeed, during the priming phase, only liver-resident CD49a^+^/DX5^−^ NK cells proliferate and confer hapten-specific CHS memory response [48], while liver CD49a^−^/DX5^+^ conventional NK cells have been shown to promote CTL anti-HBV activity via IFN-*γ* secretion, likely induced by dendritic cell- (DC-) derived IL-12 [49]. Surprisingly, during the effector phase of memory, CD49a^−^/DX5^+^ NK cells increased both in the liver and at the site of second exposure. This observation raised the possibility that liver-resident CD49a^+^/DX5^−^ NK cells may change their phenotype into CD49a^−^/DX5^+^ NK cells or alternatively they may directly or indirectly induce proliferation of CD49a^−^/DX5^+^ NK cells [48].

Exogenous substances introduced into the body from outside through gastrointestinal absorption are first transported by the bloodstream to liver, which senses and metabolizes the absorbed nutrients and chemical molecules. In the liver, hepatic immune cells directly participate in the control and elimination of potentially dangerous biological and chemical material [50]; thus, it is possible that the antigenic activation of NK cells occurs directly in that organ [48, 51]. Indeed, bacterial products, toxins, and nutritional antigens have to be quickly eliminated by innate immune cells, before these dangerous substances could create damage at systemic level [50]. Alternatively, NK cells could be activated in draining lymph nodes by antigen presenting cells, homing thereafter in the liver. After a second antigen exposure, these hepatic memory NK cells would move from the liver to the organ where the second exposure has taken place [52] (Figure 3(c)).

## 4. Memory-Like NK Cells in Viral Infections

The role of NK cells in the control of viral infections in humans has been highlighted in some rare genetic diseases resulting from a deficiency of NK cell number. In particular, human natural killer cell deficiencies have been associated with an increased susceptibility to herpesvirus infections [53]. Interestingly, the first evidence of an antigen-specific memory-like NK cell response in viral infections has been reported in a murine cytomegalovirus (MCMV) model [54]. The m157 glycoprotein, specifically expressed by MCMV-infected cells, is recognized by the Ly49H activating receptor expressed on a murine NK cell subset [38, 55] and is able to induce a selective activation and expansion of Ly49H^+^ NK cell subset [56]. However, to fully activate NK cells, antigen receptor engagement synergizes with inflammatory cytokines. Similar to activated T cells, these effector NK cells undergo a contraction phase, during which they constitute a pool of long-lived, self-renewing antigen-specific cells (Figure 3(a)). Following a subsequent exposure to MCMV, specific memory NK cells respond more quickly and effectively than the naive NK cells [54]. Similarly to MCMV, human cytomegalovirus (HCMV) is able to induce the expansion of a NK subpopulation expressing the CD94/NKG2C activating receptor [57, 58]. The expansion of CD94/NKG2C^+^ NK cells is believed to be linked to the recognition of a ligand on HCMV infected cells [59], but unlike in the mouse, this ligand has not been identified yet (Figure 3(a)). Surprisingly, this CD56^dim^/CD16^bright^/NKG2C^+^ cell subset does not perform a specific cytotoxicity against fibroblasts infected with HCMV, as expected from memory cells. Nevertheless, when stimulated by opsonized HCMV^+^ target cells via CD16 receptor, CD56^dim^/CD16^bright^/NKG2C^+^ cells display an enhanced ability to proliferate [60] and to secrete cytokines (in particular TNF-alpha) [59, 61]. Notably, humans lacking one* KLRC2* allele that encodes NKG2C receptor had a compromised NK cell differentiation during HCMV infection, with altered adaptive response and high anti-HCMV IgG titers [62, 63]. Goodier and colleagues [63] concluded that the impairment of HCMV control by adaptive NK cells would need a higher B cell response. On the other hand, since anti-HCMV IgG-coated infected cells are able to preferentially activate adaptive NK cells, it is also possible that CD56^dim^/CD16^bright^/NKG2C^+^ functional adaptations may be “aimed” to improve the cooperation with B lymphocytes, finally leading to a lower need of anti-HCMV IgG secretion for controlling HCMV.

Increase of CD56^dim^/CD16^bright^/NKG2C^+^ NK cells has also been found in patients with other viral infections, such as hepatitis C virus (HCV), hepatitis B virus (HBV) [64, 65], EBV (Epstein Barr Virus) [66], or HIV-1 [67], but only in people previously infected with HCMV as well, suggesting that expansion of the CD94/NKG2C^+^ NK cell subset is CMV specific rather than a generalized response after virus infection. Interestingly, HCMV drives clonal-like expansions of NK cell subsets commonly characterized by increased expression not only of NKG2C, but also of self-HLA-I-specific KIRs and CD57, as well as decreased expression of Fc*ε*R*γ*, SYK, and EAT-2 adaptor molecules, which may drive the functional changes of these adaptive, memory-like NK cells [19, 60, 68, 69]. Indeed, HCMV induces epigenetic modification of signaling molecules in adaptive NK cells that affect their effector function [60].

Moreover, NK cells with memory characteristics have been found after exposure to viral-like particles or in various viral infections such as genital herpes (HSV-2), vaccinia virus, influenza, and human immunodeficiency virus type 1 (HIV-1) [47, 70–73]. A murine study has demonstrated the existence of short-term antigen-specific NK memory against HSV-2 virus. Following the reexposure to the same viral antigen, memory-like NK cells are able to respond with an increased IFN-*γ* production [70]; nevertheless they do not acquire NKG2C antigen expression [71], confirming again the peculiar association of CD94/NKG2C^+^ subset with CMV infection. Finally, poxvirus infection has also been described to induce an immunological NK memory response in mouse. A subset (Thy1^+^) of NK cells, generated in a first exposure to infection, are sufficient to provide protection against lethal doses of virus injections and, similarly to CHS model, reside in liver [72]. Differently from vaccinia virus- and CHS-induced memory-like NK cells, after influenza virus infection the primary site of proliferation of long-lived NK cells is the bone marrow [73]. Since influenza-induced long-lived NK cells proliferate even after respiratory syncytial virus challenge, the authors conclude that cytokine activation alone is likely generating these long-lived NK cells and that the bone marrow is not only a site of NK differentiation but also an important site for homing of memory-like NK cells [73].

Although in the majority of these viral infection models NK cells are thought to protect host in an antigen-specific manner, the NK cell proliferative signals produced by proinflammatory cytokines IL-18, type I-IFN, and in particular IL-12 are also indispensable in the generation of virus-specific NK cell memory [74, 75]. Downstream of inflammatory signals, Zbtb32 transcription factor has been shown to have an essential cell-intrinsic function to generate NK cell memory [76]. Intriguingly, it has been also described that a subset of NK cells transiently expressed RAG during their development and its expression is critical for their survival following virus infection, indicating a cell-intrinsic role for RAG in NK cells. This subset of NK cells is responsible for more robust memory response to MCMV infection [77], suggesting an involvement of RAG during the development of a NK cell subset specialized in the generation of MCMV-specific memory-like NK cells.

## 5. Antigen-Independent Long-Lived Cytokine-Activated NK Cells: Involvement in Trained Immunity and Vaccine

Numerous experimental evidences suggest that the stimulation of NK cells with proinflammatory cytokines is able to generate long-lived activated NK cells. Such NK cells activated in vitro with IL-12, IL-15, and IL-18 and then inoculated in* Rag1* ^−*/*−^ mice produce higher amount of IFN-*γ* after a subsequent restimulation with cytokines. This feature is maintained for at least 12 weeks after inoculation and seems to be an intrinsic characteristic of the cytokine-activated NK cells that can be transmitted to the progeny [78, 79]. Similar cytokine-induced memory-like NK cells were also observed in both human CD56^dim^ and CD56^bright^ NK cell subsets [80]. The increased capacity to produce IFN-*γ* and the prolonged longevity of these long-term activated NK cells induced by proinflammatory cytokines seem to be independent of the recognition of a viral antigen by activating receptors. Therefore, these cytokine-induced long-lived activated NK cells show an antigen-independent functional memory, which is distinct from that induced by CMV specific activating receptors (Figure 3(b)).

Nevertheless, similar to HCMV-induced CD94/NKG2C^+^ NK cells and memory type 1 T helper cells [60, 81], an exposure of human NK cells to IL-12, IL-15, and IL-18 resulted in NK cell stable demethylation of the IFNG locus regulatory regions [81], suggesting that epigenetic imprinting is a common hallmark driving both antigen-dependent and antigen-independent memory-like NK cells.

Both CD56^dim^ and CD56^bright^ NK cell subsets, preactivated with IL-12, IL-15, and IL-18, showed an increased production of IFN-*γ* (but not degranulation) to a subsequent stimulation with cytokines or with the HLA-I-negative K562 leukemic cell line [80], suggesting new potential approaches in NK cell-based immunotherapies against cancer. A characteristic of these memory-like cells is the prolonged expression of CD25, which confers responsiveness to low amounts of IL-2 [82], thus indirectly enhancing NK cell response to IL-2 produced by CD4^+^ T cells. These observations have provided the rationale for the immunotherapeutic strategies based on adoptive cell transfer of cytokine preactivated NK cells, followed by low dose IL-2 administration. Remarkably, in some patients with acute myeloid leukemia, clinical responses after adoptive transfer of long-lived cytokine-activated cells have been described [83]. In a recent study it has also been shown that, after annual flu vaccination, NK cells were more prone to produce IFN-*γ* and also to provide a response against other influenza strains different from the vaccine, ensuring a wider coverage and a vaccine-induced cross-protection for several months [84]. This response is reminiscent of the phenomenon termed “trained immunity,” first described in monocytes. According to this phenomenon, monocytes respond with an increased cytokine production upon restimulation due to epigenetic changes induced by the first stimulation or priming [85, 86]. Similar to long-lived cytokine-activated NK cells, the improved responsiveness to subsequent challenges coincided with their epigenetic changes and was independent of the nature of restimulation. As a matter of fact, this kind of response has also been described in NK cells. Interestingly, in mice lacking B and T cells, priming of NK cells with Bacillus Calmette-Guérin (BCG) gave protection against a wide spectrum of pathogens [87]. In line with these observations is the employment of BCG as adjuvant in cancer therapy [88]. Indeed, BCG has been shown to induce* in vivo* antitumor effects via DC toll-like receptor 2 (TLR2) and TLR4 [89] and proinflammatory cytokines produced by DC in response to TLRs likely represent the explanation of anticancer mechanisms induced by microbial components [28].

**Figure 4:**
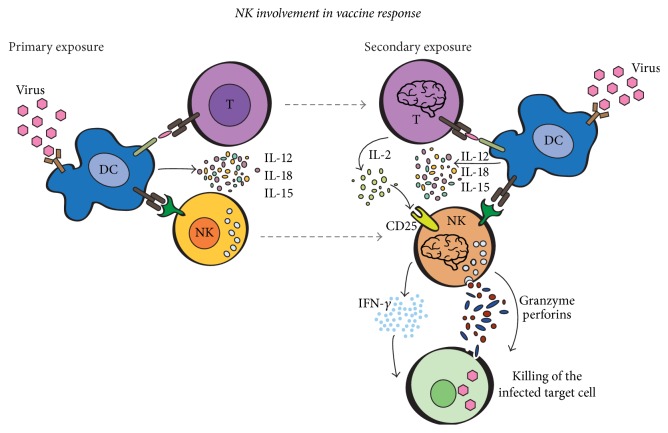
Memory-like NK cell involvement in vaccination. During the rabies virus vaccination (primary exposure), IL-15, IL-12, and IL-18, produced by virus activated dendritic cells (DCs), generate T and long-lived cytokine-activated NK cells. In the postvaccination recall (secondary exposure), IL-15, IL-12, and IL-18 together with IL-2 produced by memory T cells would induce a strong degranulation and an intense IFN-*γ* secretion by long-lived cytokine-activated NK cells.

Interestingly, NK cells appear to play a role in the control of rabies virus infection after vaccination. As a matter of fact, after in vitro PBMC stimulation with inactivated rabies virus, only NK cells in vaccinated individuals showed a strong and prolonged cytokine (IFN-*γ*) production and degranulation [90]. NK cells represent the main population of cells able to secrete IFN-*γ* and to degranulate in the first 12–18 hours after reexposure to the inactivated virus, indicating that they are necessary for a rapid response after vaccination. During the first exposure to the virus, the interaction of the virus with antigen presenting cells (APC) would induce IL-12, IL-18, and also IL-15 secretion which would prime not only naïve T, but also NK cells into long-lived CD25^+^ NK cells [82]. During the reexposure of the virus, the intense NK cytokine production and the potent NK degranulation would be also determined by the synergy of proinflammatory cytokines from APC with IL-2 from antigen-specific CD4^+^ T cells (Figure 4).

Similarly, it has been shown that the response of T cells induced by a HIV-1 specific vaccine is able to improve NK cell function, typically impaired in individuals with HIV-1 chronic infection [91]. However, vaccination against specific viral antigens (influenza, vesicular stomatitis virus, or HIV-1) has been shown to generate not only long-lived cytokine-activated NK cells but also memory-like NK cells capable of protecting mice against viral challenge in a virus/antigen-specific manner [47]. The data indicate that NK cells are important actors in vaccine-induced immune responses and assessment of NK cell activation after vaccination could represent a further and important indicator of vaccine efficacy.

## 6. Concluding Remarks and Future Research

NK cells, as well as monocytes, have historically been considered short-lived, rapid, and aspecific effectors of the innate immunity; however, it is now clear that, once sensitized by viral antigens or haptens, they are also able to mount an adaptive response that resembles the classical immunological memory [75]. In fact, challenging the classical concept of immunological memory, NK cells with an extended lifespan and an enhanced recall response have been characterized. NK cells can respond to antigens although with a limited choice of antigen specificity. The specific interaction between the glycoprotein m157 and Ly49H receptor makes the MCMV infection model a special case for the NK cell memory. This NK antigen recognition resembles that of T cell memory generation, since Ly49H^+^ memory-like NK cells require costimulatory signals. In fact, Ly49H^+^ NK cells missing the DNAM-1 receptor fail to expand and to form long-lived memory NK cells [92].

### 6.1. Cooperation of NK Cells with B Cells

Differently from mice, in humans it is not clear whether the expansion of CD56^dim^/CD16^bright^/NKG2C^+^ NK cells is aimed at protecting against a second exposure to the virus or rather represents a “side effect” of HCMV infection. Of note, CD56^dim^/CD16^bright^/NKG2C^+^ NK cells develop a specific adaptive feature [59–61] characterized by an enhanced response via CD16 stimulation, which suggests an improved cooperation of memory-like NK cells with B cells. In line with this hypothesis, the T cell cytokine IL-21 has been described to induce both the development of CD56^dim^/CD16^bright^ NK cells from CD34^+^ hematopoietic progenitors [12, 13, 93] and the switching of Ig produced by B cell to IgG1 and IgG3 isotypes [94], for which Fc*γ*RIIIA receptor (CD16 of NK cells) displays a higher affinity [95]. Thus, through IL-21 secretion, T cells would be able to coordinate an ADCC response, inducing both an adequate isotype switching in B cells and an ADCC specialized NK cell subset. Similarly, other T cell cytokines (i.e., IL-2 and/or type 2) might be involved in the generation of memory-like NK cells with a peculiar understanding with B cells.

### 6.2. Sites of Memory NK Cell Generation and Homing

Although there are several evidences of human NK antigen recognition and generation of memory-like NK cells, many gaps remain in understanding the mechanisms, particularly the sites and the receptors involved in these processes. Both HCMV and MCMV memory-like NK cells are not organ-specific, while in CHS model and in some viral infection memory-like NK cells are resident in the liver [46–48, 51, 72]. For this reason, it has been suggested that in CHS model the antigen recognition by NK cells could occur in the liver [48, 51]. On the other hand, the bone marrow is the primary site of long-lived NK cells generated after influenza infection [73]. The homing of memory-like NK cells might be imprinted by the first site of NK antigen/hapten recognition, consequently depending on the NK cell subset involved, on the way of entering into the body, and on the organ for which the antigen/hapten expresses its tropism.

### 6.3. Are NK-LGLs Memory-Like NK Cells?

How human NK cells could recognize antigens is under debate. A possibility is suggested by the observation that viral peptides can interfere with MHC-I recognition by NK inhibitory receptors [37], unleashing NK cell response in a “masking self-recognition” fashion. On the other hand, activating NK cell receptors that selectively recognize viral peptides/antigens mounted on MHC-I or HLA-E have been associated to NK memory-like and NK-LGL expansion [54, 57, 96]. Intriguingly, HCMV-driven CD94/NKG2C^+^ NK clonal expansion shows several similarities with NK-type chronic lymphoproliferative disease of granular lymphocytes (LGL). NK-LGL is an indolent NK cell disease characterized by a persistent increase of circulating NK cells, typically expressing activating forms of KIRs [97]. Remarkably, in the large majority of NK-LGL patients, there is a serologic evidence of past viral infection, suggesting that viral infection and collateral proinflammatory cytokine secretion may play a role early in disease pathogenesis [97, 98]. Intriguingly, NK-LGL positive for KIR3DS1 are mainly confined to HLA-Bw4 positive individuals [99, Zambello R, unpublished data], suggesting that KIR3DS1^+^ clonal expansion might be driven by (viral) peptides mounted on HLA-Bw4 molecules. Therefore, the NK-LGL could be a good “model” to explore in order to shed more light to NK clonal expansion. Moreover, a heavy methylation of inhibitory KIR3DL1 promoter was also described in NK-LGL [100]. The authors conclude that the lack of the inhibitory signal together with the increased expression of activating receptors could play a role in the disease pathogenesis. On the other hand, epigenetic modifications are typical of both adaptive memory T and memory NK cells and have been described in both virus-specific and cytokine-induced memory-like NK cells [60, 75, 81]. Thus, a more detailed understanding of the epigenetic control could help to better understand the mechanisms of chronic lymphoproliferative diseases and lead to new NK cell-based immunotherapeutic approaches.

### 6.4. Are TLRs Involved in NK Memory Formation?

Of note, the limited antigen specificity of activating Ly49H (and likely CD94-NKG2C) receptors against pathogen ligands is not so far from that of TLRs, an old and evolutionarily conserved system for recognition of pathogen-associated molecules and danger-associated molecular patterns [89]. Similar to Ly49H and CD94-NKG2C receptors, these innate antigen-specific receptors require the presence of accessory cells or of proinflammatory cytokines to induce NK cell responses [101]. Moreover, resembling NK cell response to cytokines [15, 16], there are evidences of heterogeneity in the ability to respond to TLRs [102], further confirming that NK cell subsetting is an intrinsic characteristic of NK cell lineage. Whether or not TLRs are involved in NK memory-like formation is not documented and evidences for innate immune memory (basically, trained immunity) associated with TLRs are limited [75]. Nevertheless, distinct TLRs have been associated with a specific protection against several viral infections [101], raising the possibility that some virus-specific memory-like NK cells could be driven by a specific TLR engagement. Indeed, TLR2 signaling on NK cells, but not on accessory cells, has been shown to be necessary for efficient NK cell activation and control of vaccinia virus infection in vivo [103]. Moreover, a report has highlighted a prominent role of TLRs in contact allergen-induced innate immune activation [104]. The authors describe a hapten-specific CHS selectively mediated by TLR2 or TLR4 [104]. These evidences are suggestive of a role of TLRs in the hapten-specific memory-like NK cell-generation. If this is the case, the hapten-specific NK cell response should be directly mediated by NK TLRs rather than DC ones. In fact, TLR-activated DCs would, in turn, produce a general hapten-non-specific inflammation.

From an evolutionary point of view, TLRs are the prototypes of the antigen specific receptors. It is tempting to speculate that the main difference between the two types of activating receptors lies in the MHC-I “intermediation.” NK cells are known to be the first lymphoid cells to appear during ontogenesis and phylogenesis and TLRs one of the first activating receptors expressed during NK cell “evolution.” Activating receptors recognizing MHC-I molecules/peptides evolved later, together or after the occurrence of MHC-I molecules either on an ancient NK cell population or on a new evolved NK subset.

### 6.5. Heterogeneity of the NK Cell Population

Whether memory-like NK cells can develop from all or only a subset of NK cells is currently not clear. However, several observations suggest the possibility that different NK cell subsets act against distinct pathogens or in a different manner. This is attested by the recent evidence that mouse Ly49H^+^ and Ly49H^−^ NK cell subsets give a complementary protection to MCMV differentiating into memory-like and cytokine-activated NK cells, respectively [105]. A similar behavior has been reported both for mouse liver CD49a^+^/DX5^−^ and CD49a^−^/DX5^+^ and for human peripheral blood CD56^dim^/CD16^bright^ and CD56^bright^/CD16^dim/neg^ NK cell subsets. In line with this, there is also the observation that RAG expression during NK cell ontogeny correlates with functionally distinct progeny cells, indicating heterogeneity in the NK cell population (see review [19]), as, in general, in ILC groups. Remarkably, there are recent evidences of innate lymphoid cell redundancy [106]. It is possible that during the evolution of the immune system some ILC subsets, originally developed against a specific pathogen, have lost their usefulness in the current (relatively “aseptic”) environment (“hic et nunc”). Nevertheless, the immune system and life in general maintain trace of their existence; they do not forget the past, their history, perhaps because memory is an intrinsic characteristic of the immune system and, in general, of the current life, likely selected among the primordial types of life. This could suggest that they are not eliminated during evolution because might they become useful again in the future?

### 6.6. Walking Four Paths along Ontogenetic Memory Lane

**Figure 5:**
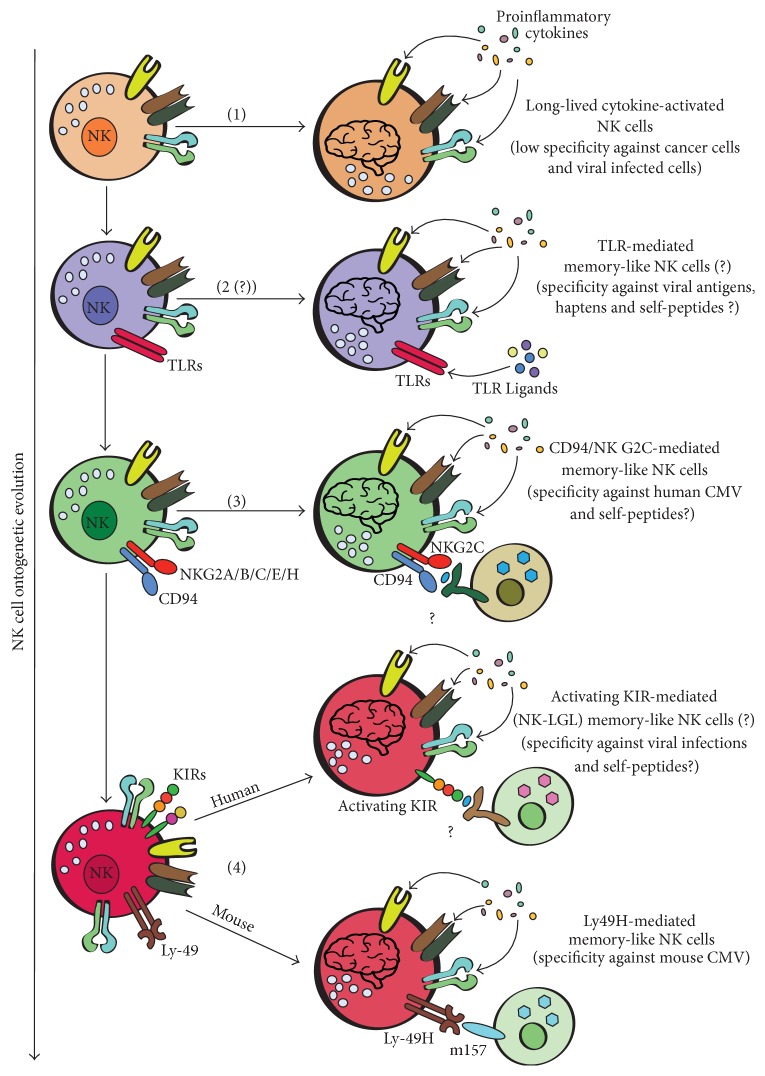
Along four pathways of NK cell memory. Exploiting the occurrence of different activating receptors surfaced during NK cell evolution, NK cells have developed different abilities to generate NK cell memory. A model with the sequence of the different NK cell “memories” occurred during NK cell ontogenesis is proposed. TLRs: toll-like receptors; NK-LGL: NK-type lymphoproliferative large granular lymphocytes.

Altogether the data would suggest that while B and T cell are preferentially able to mount an immunological response inducing modification within a relatively homogeneous population (differing mainly for the conformation of one receptor), NK cells and in general ILCs along their ontogeny have privileged the expansion of different cell populations with distinct functional features. We suggest that this NK cell heterogeneity constitutes also the basis of the different types of NK cell memories. Based on NK cell receptors and/or subsets involved in NK memory-like formation, a sequence of four different types of NK cell memory occurring during NK cell evolution could be hypothesized: (1) long-lived cytokine-activated NK cells (low specificity, possibly limited to specific cytokine-responder NK cell subsets); (2) TLR-mediated memory-like NK cells (perhaps against some viral antigens and haptens); (3) CD94/NKG2C-mediated memory-like NK cells (specific against HCMV); (4) activating Ly49-mediated memory-like NK cells and activating KIR-mediated (NK-LGL) memory-like NK cells (specificity against MCMV and perhaps some viral infections, resp.) (Figure 5). A similar sequence of NK cell types could be also imagined for NK cell-mediated autoimmune diseases, in which some activating KIRs have been associated with an increased susceptibility [36, 107]. In this case, instead of foreign, self-antigens or peptides (mounted or not mounted on MHC-I) are supposed to drive an anomalous recognition by activating KIRs and perhaps TLRs expressed by NK cell subsets (Figure 5).

### 6.7. Do NK Cells Protect the Species from New Evolved Pathogens?

Interestingly, in some subjects, depending on the type of disease and on the relationships between KIRs and their ligands, NK cells can function as well as CTLs, producing a long-lasting immune cell response against specific viral infections, neoplastic transformation, and autoimmune disorders. These evidences suggest a possible complementary and nonredundant role shared by T and NK cell populations (and ILCs in general), the first preferentially aimed to protect single members of a species from “known” pathogens and the second, thanks to their interindividual heterogeneity, preferentially aimed to protect the species as a whole from new evolved pathogens. It is well known that in the single individual NK cells rapidly tackle and predispose to the development of following immune-adaptive eradication of “known” pathogens. It is tempting to speculate that similarly, in the occurrence of new pathogens evolved under the strong selective pressure produced by the immune (adaptive) system, within the whole population the interindividual heterogeneity of NK cells gives more chance to temporarily protect the species (allowing reaching the reproductive age), finally giving the time to develop a new specialized and stronger adaptive immunity, which will become heritage of the following generations. The majority of the scientific studies that evaluate the performance of the different immune populations are performed in the current relatively “static” environment and do not take in account this evolutionary scenario. For this reason, the actual impact and importance of this heterogeneous population of cells has been for a long time underestimated. On the other hand, in a more “dynamic” and challenging environment, as in the case in which a population faces an infectious agent never met before, the impact of NK cell response has been suggested to increase [25, 26, 108].

### 6.8. Conclusive Considerations

The rapidity of NK cell response could be exploited to exert an early control of infections, and in case of HIV, which target cells of the adaptive immune system, it could be finalized to prematurely limit the spread of the virus. The fact that NK cells acquire a “memory” broad spectrum, able to act also against pathogens or transformed cells not involved in the first activation, has opened new horizons in the development of next-generation vaccines and in therapeutic use of long-lived activated NK cells against cancer. Further studies are required to elucidate the mechanisms involved in the generation and maintenance of the memory-cell pools, to identify the specific markers expressed by these memory NK subsets, and to understand the pathways involved in antigen recognition, not to speak of the molecular epigenetic mechanisms behind the generation of memory-like NK cells.
